# Survival Outcomes of Patients With Epidermal Growth Factor Receptor Mutations in Non-Small Cell Lung Cancer With Leptomeningeal Metastasis

**DOI:** 10.3389/fonc.2021.723562

**Published:** 2022-01-20

**Authors:** Ning Li, Zhimin Bian, Minghua Cong, Yutao Liu

**Affiliations:** ^1^ Department of Comprehensive Oncology, National Cancer Center/National Clinical Research Center for Cancer/Cancer Hospital, Chinese Academy of Medical Sciences & Peking Union Medical College, Beijing, China; ^2^ Department of Medical Oncology, National Cancer Center/National Clinical Research Center for Cancer/Cancer Hospital, Chinese Academy of Medical Sciences & Peking Union Medical College, Beijing, China

**Keywords:** survival analysis, EGFR-mutated, NSCLC, leptomeningeal metastasis, CSF

## Abstract

**Background:**

Leptomeningeal metastasis (LM) is a commonly observed complication in patients with epidermal growth factor receptor (EGFR)-mutated non-small cell lung cancer (NSCLC). This study aimed to investigate the gene mutations, treatment strategies, and clinical outcomes in patients with LM.

**Methods:**

We retrospectively analyzed the clinical and survival outcomes of 53 patients with *EGFR*-mutated NSCLC with LM.

**Results:**

The median overall survival after LM diagnosis was 13.0 months, ranging from 0.5 to 42.0 months (95% CI = 9.067–16.933), with 64.2% maturity. Patients who received osimertinib after developing LM (n = 35) had a significantly higher rate of LM disease control (*p* = 0.008) and significantly longer overall survival (15.0 *versus* 6.0 months; hazard ratio (HR), 2.4292; 95% CI, 1.234–4.779; *p* = 0.045) than those who received previous generations of EGFR tyrosine kinase inhibitors (TKIs) or other localized therapies (n = 6). Logistic regression analysis showed that LM disease control status was a positive predictive factor for overall survival after developing LM (*p* < 0.001, odds ratio = 10.797, 95% CI = 4.102–28.419).

**Conclusions:**

Our study provides real-world clinical evidence that patients with *EGFR*-mutated NSCLC diagnosed with LM who developed LM had better clinical outcomes with osimertinib therapy. Our findings also suggest that LM disease control is the most effective strategy to prolong the overall survival outcomes of these patients.

## Introduction

Non-small cell lung cancer (NSCLC) accounts for almost 85% of lung cancers (1). The survival rate of patients diagnosed with lung cancer has improved along with the advances in detection methods and the availability of targeted therapies. Leptomeningeal metastasis (LM) is a devastating complication of advanced lung cancer, with an incidence of 5%–9% (2, 3). Patients with NSCLC who harbor sensitizing mutations in the epidermal growth factor receptor (EGFR) were more likely to develop LM (4). One of the primary explanations for the increased frequency of LM for this molecular subgroup is their more prolonged overall survival with EGFR tyrosine kinase inhibitor (TKI) therapy (5). Moreover, this molecular subgroup would inevitably develop secondary resistance to EGFR TKIs, and one of the possible sites of progression is the central nervous system, including the leptomeninges (6–8). Previous studies have reported that first- and second-generation EGFR TKIs have poor penetration across the blood–brain barrier, with the percentage of drug penetration ranging between 0.7% and 1.3%, which may permit tumor growth in the central nervous system (9).

There are currently three main therapeutic approaches in the management of LM: systemic chemotherapy and two localized therapies, intrathecal chemotherapy (ITC) and whole-brain radiation therapy (WBRT). The optimal treatment method remains elusive, and no treatment strategy has been considered as standard of care. Hence, LM prognosis remains poor, with a median survival of only 3 months in molecularly unselected NSCLC patients (10).

With the increased use of newer generation of EGFR TKIs, survival outcomes of patients with *EGFR*-mutated NSCLC with LM have been extended up to 10 months (4, 11). However, clinical studies at LM diagnosis that include paired blood and cerebrospinal fluid (CSF) mutational status are limited. In this retrospective study, we report on clinical and survival outcomes and mutational status of patients diagnosed with *EGFR*-mutated NSCLC who developed LM.

## Materials and Methods

### Study Design And Patient Cohort

This retrospective study included 53 patients with *EGFR*-mutated stage IV NSCLC with LM who were treated in the general department or internal medicine of our hospital due to neurological symptoms between January 2016 and April 2021. All the patients were diagnosed by CSF cytology for the presence of malignant cells and/or MRI. The Eastern Cooperative Oncology Group Performance Status (ECOG PS) was evaluated for each patient at LM diagnosis. Medical data for these patients were reviewed. Paired CSF and blood samples were collected from patients at LM diagnosis. CSF samples measuring 10 ml, collected by lumbar puncture, and 8 ml of plasma samples from each patient were submitted for gene testing using either amplification refractory mutation system (ARMS) or next-generation sequencing (NGS). *EGFR* mutations included were exon 18, exon 19 deletion, exon 21 L858R, exon 20, and T790M. The *EGFR* mutation status of all patients was confirmed by targeted NGS analysis of tumor DNA extracted from primary tumor or metastatic tumor tissue samples collected at initial diagnosis of NSCLC and cell-free DNA (cfDNA) extracted from paired blood and CSF samples collected at LM diagnosis (Burning Rock Biotech, Guangzhou, China). The ethics committee of the Cancer Hospital, Chinese Academy of Medical Sciences, and Peking Union Medical College approved the protocol.

### Assessments

Follow-up of all the patients was carried out until May 2021. The duration of investigation was calculated from the time of LM diagnosis to death or the last date of follow-up, with a minimum follow-up period of 1 month for inclusion in statistical analysis. Disease control for LM was assessed using these two criteria: LM is assessed as “improved/stable” when the palliation of clinical symptom is achieved, and/or MRI examination showing a decreased or stable lesion, while LM is assessed as “worse” when the clinical symptoms worsen or lesions were observed to increase on MRI examination, according to the clinical practice guidelines recommended by the European Association of Neuro-Oncology–European Society for Medical Oncology (EANO–ESMO) for managing patients with LM from solid tumors. Four weeks after LM diagnosis, extracranial lesions that appeared to be LM were evaluated according to Response Evaluation Criteria in Solid Tumors (RECIST) version 1.1 and categorized as complete response (CR), partial response (PR), stable disease (SD), or progressive disease (PD). The primary outcome for this study was overall survival after LM diagnosis (OS_LM_), calculated from the date of LM diagnosis to the date of death.

Survival analyses were performed using the Kaplan–Meier method and the Gehan–Breslow–Wilcoxon test of significance. Subgroup comparisons were performed using Cox proportional hazards model and Wald 95% CIs. *p*-Values less than 0.05 were considered statistically significant. Statistical analyses were performed using SPSS for Windows (version 22; SPSS Inc., Chicago, IL, USA) and GraphPad Prism 8 (La Jolla, CA, USA). The analysis cutoff date was May 31, 2021.

## Results

### General Characteristics of Patients With *EGFR*-Mutated Non-Small Cell Lung Cancer Diagnosed With Leptomeningeal Metastasis

Among the 53 patients included in our cohort, LM was confirmed by CSF cytology in 50 patients and MRI in three patients. There were slightly more female than male patients (30/53; 56.6%). The median time from NSCLC diagnosis to LM diagnosis was 18.0 months (range: 0–88). Most patients (37/53; 69.8%) had brain metastasis (BM) before or simultaneously with LM diagnosis. The ECOG PS was 0–2 for 41.5% of patients, with most extracranial tumors evaluated as SD or PR at LM diagnosis (37/53; 69.8%). Before LM diagnosis, three patients had history of WBRT (5.7%), and 30 (56.5%) patients had history of cytotoxic chemotherapy. A majority (n = 47, 88.7%) of the patients received EGFR TKI therapy before developing LM, while the five remaining patients were EGFR TKI-naïve. **Table 1** summarizes the characteristics of our cohort.

**Table 1:** Clinical characteristics of patients with *EGFR*-mutated advanced non-small cell lung cancer (NSCLC) with leptomeningeal metastasis (LM) included in this study.

Characteristics	N = 53 (n, %)
Median age, years (range)	56 (23–70)
Sex (male/female)	
Male	23 (43.4%)
Female	30 (56.6%)
Time from NSCLC diagnosis to LM, months (range)	18 (0–54)
Histology	
Adenocarcinoma	53 (100%)
Eastern Cooperative Oncology Group Performance Status	
0–2	22 (41.5%)
3–4	31 (58.5%)
Extracranial disease objective response at LM diagnosis	
SD/PR	37 (69.8%)
PD	16 (30.2%)
Brain metastasis status	
With (before or concurrent with LM diagnosis)	37 (69.8%)
Without	16 (30.2%)
Tissue-based *EGFR* mutational status before LM	
* EGFR* exon 19 deletion	19 (35.8%)
* EGFR* L858R	26 (49.1%)
* EGFR* exon 20 insertion	2 (3.8%)
* EGFR* L861Q	1 (1.9%)
Not tested	5 (9.4%)
History of brain radiotherapy before LM	
Whole brain	3 (5.7%)
Other	3 (5.7%)
Without	47 (88.7%)
History of cytotoxic chemotherapy before LM	
With	30 (56.6%)
Without	23 (43.4%)
History of EGFR TKI therapy before LM	
First-generation	29 (54.7%)
Second-generation	4 (7.5%)
Third-generation	14 (26.4%)
Pyrotinib	1 (1.9%)
EGFR TKI-naïve	5 (9.4%)

ERBB2, Erb-B2 receptor tyrosine kinase 2; EGFR TKI, epidermal growth factor receptor tyrosine kinase inhibitor; LM, leptomeningeal metastasis; PD, progressive disease; SD/PR, stable disease or partial response according to RECIST definitions of response to treatment.

### Mutational Status of DNA From Tissue, Cerebrospinal Fluid, and Blood

Targeted sequencing was performed on paired CSF and blood samples collected at LM diagnosis from patients with either *EGFR* exon 19 deletion (19del) (n = 19) or *EGFR* exon 21 L858R (n = 26) detected from their tumor samples at baseline (before developing LM).

**Figure 1:**
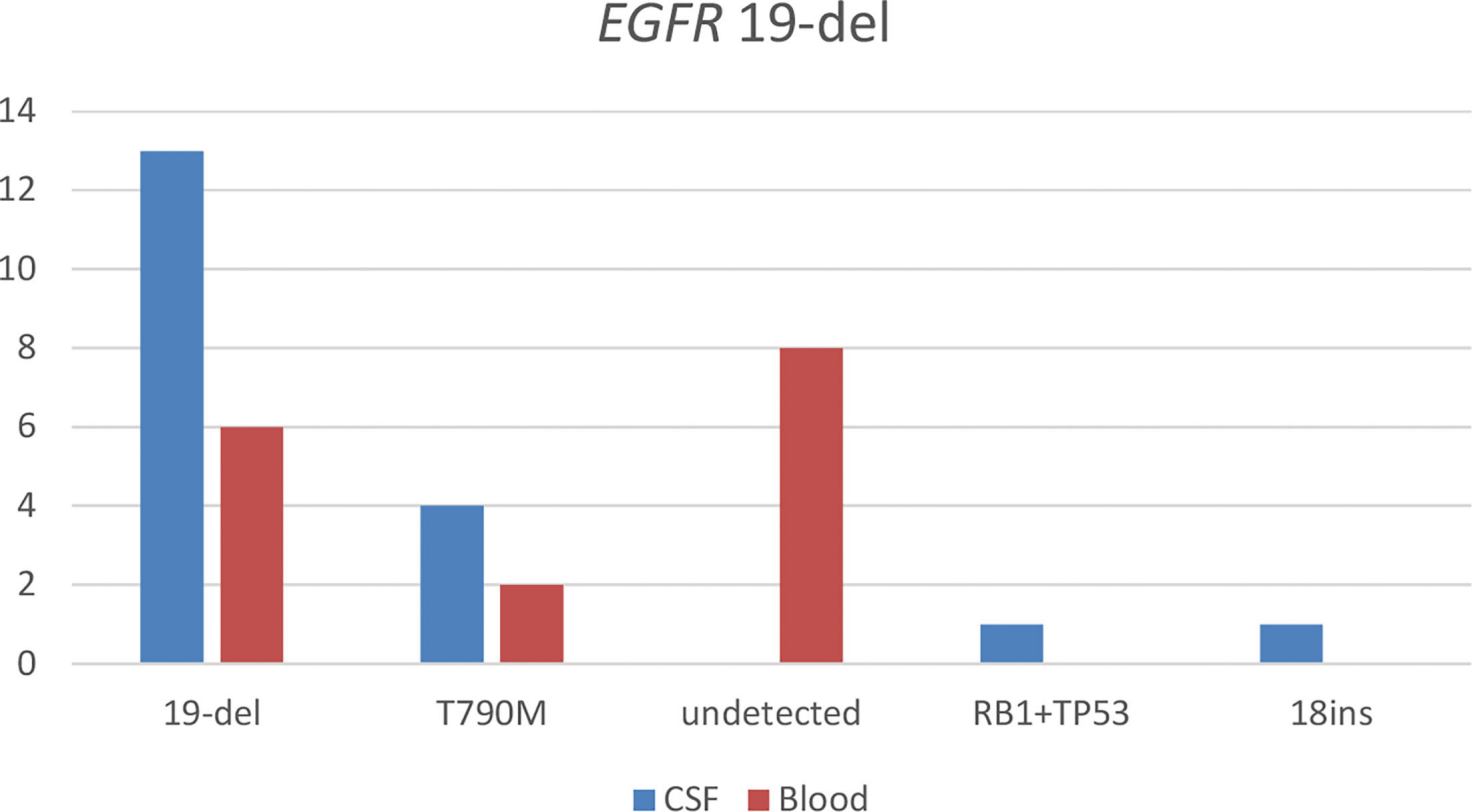
Detection of somatic mutations in paired cerebrospinal fluid (CSF) and blood samples of patients with leptomeningeal metastasis (LM) harboring *EGFR* exon 19 deletion (19del) detected from tissue samples before LM diagnosis. 18ins, *EGFR* exon 18 insertion; RB1+TP53, a mutation in retinoblastoma 1 and tumor protein p53.

With the use of the paired CSF samples of 19 patients having baseline *EGFR* 19del mutation detected from their tissue samples, 68.4% (n = 13) of the patients were detected with *EGFR* 19del at LM diagnosis. *EGFR* T790M was detected in 21.1% (n = 4), with two patients having concurrent *EGFR* 19del. *EGFR* 19del concurrent with retinoblastoma 1 (*RB1*) was detected in one patient, tumor protein p53 (*TP53*) mutations (n = 1) in one patient, and *EGFR* 19del concurrent with *EGFR* C797S (n = 1) in one patient. *EGFR* exon 18 insertion mutations were detected in one patient. *EGFR* mutations were not detected in CSF samples of three patients. With the use of their paired plasma samples, *EGFR* 19del was detected in only 31.6% (n = 6). *EGFR* T790M was detected in 10.5% (n = 2), with a patient detected with concurrent *EGFR* 19del. Eight of the patients were not detected with any *EGFR* mutations from their plasma sample. **Figure 1** illustrates the results from this analysis.

**Figure 2:**
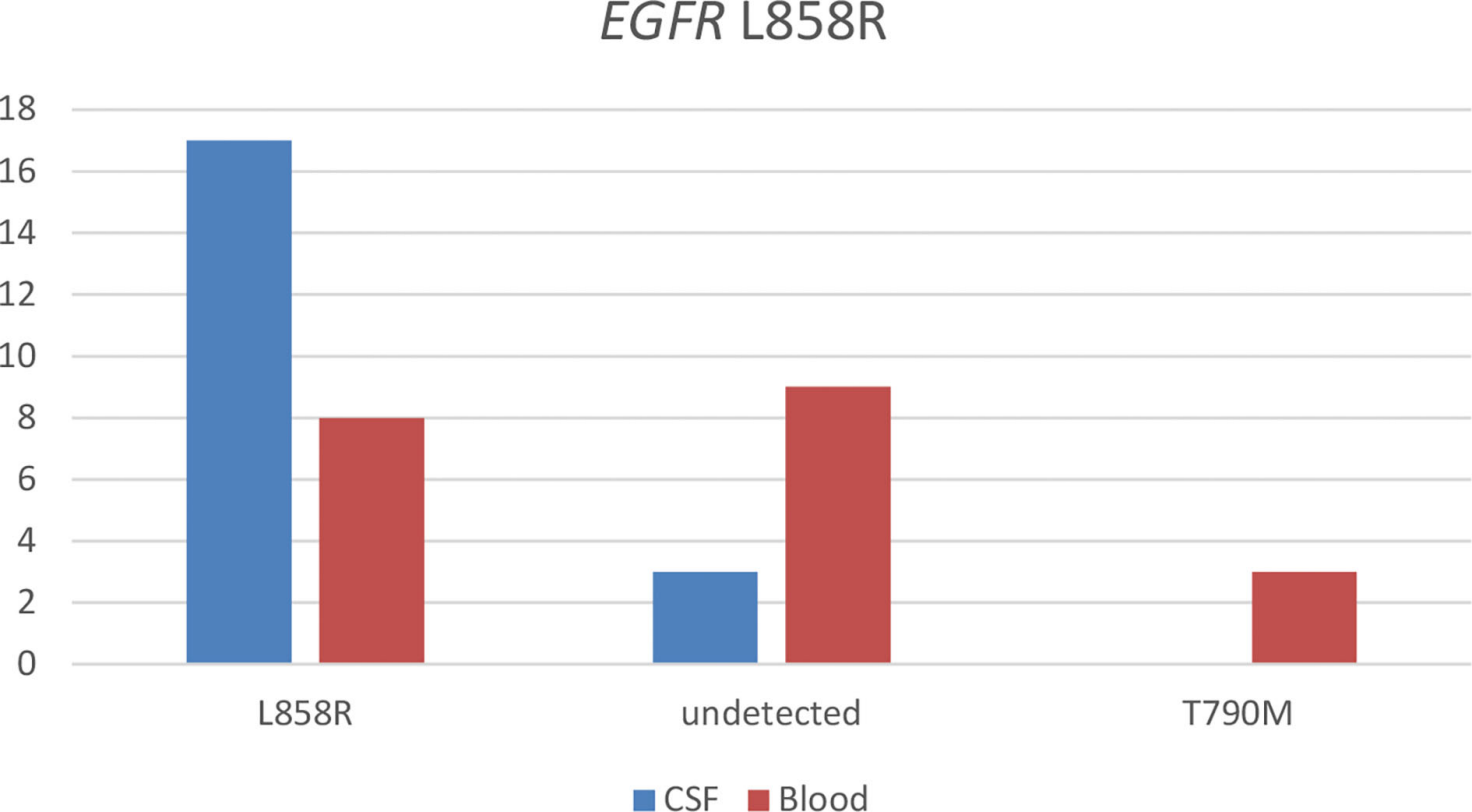
Detection of somatic mutations in paired cerebrospinal fluid (CSF) and blood samples of patients with *EGFR*-mutated non-small cell lung cancer (NSCLC) diagnosed with leptomeningeal metastasis (LM) harboring *EGFR* L858R detected from their tissue sample before LM diagnosis.

With the use of the paired CSF samples of 26 patients having baseline *EGFR* L858R detected from their tissue samples, *EGFR* L858R was detected in 65.4% (n = 17) of their plasma sample at LM diagnosis. *BRAF* mutation (n = 1) and *MET* amplification (n = 1) were detected in a patient each. Three patients (11.5%) were not detected with any *EGFR* mutations from their CSF samples. With the use of their paired plasma samples, *EGFR* L858R was detected in 30.8% (n = 8). *EGFR* T790M was detected in 11.5% (n = 3), *EGFR* exon 19 p.V7421 (n = 1) in one patient, *MET* amplification (n = 1) in one patient, and *HER2* mutations (n = 1) in one patient. Nine patients (34.6%) were not detected with any *EGFR* mutations in their plasma samples. **Figure 2** illustrates the results from this analysis.

Taken together, these results demonstrate that somatic mutations, particularly *EGFR* mutations, were more likely to be detected in CSF samples than in plasma samples, indicating that CSF samples are the optimal tumor DNA source for molecular testing in patients with LM.

### Efficacy of Treatment in *EGFR*-Mutated Non-Small Cell Lung Cancer Patients After Leptomeningeal Metastasis

Of the 29 patients who received first-generation EGFR TKIs before developing LM, four of the five patients who continued to receive first-generation EGFR TKIs (i.e., gefitinib and erlotinib) after developing LM achieved LM disease control. Of the 24 patients who received osimertinib after developing LM from first-generation EGFR TKI, 23 patients achieved LM disease control. Of the four patients who developed LM with afatinib therapy, two of the three patients who received osimertinib after LM diagnosis achieved LM disease control, while one patient did not receive any EGFR TKIs. Of the 14 patients who received third-generation EGFR TKIs (i.e., osimertinib and zorifertinib/AZD3759) before developing LM, 12 continued to receive osimertinib, and two did not receive any EGFR TKIs treatment after LM diagnosis. Of the 12 patients who continued to receive osimertinib after developing LM, eight patients achieved LM disease control. One patient developed LM during pyrotinib (a dual EGFR/HER2 TKI) therapy and did not receive EGFR TKIs after LM diagnosis. Among the five patients who were EGFR TKI-naïve before their diagnosis with LM, two patients were treated with osimertinib, and both achieved LM disease control. **Table 2** summarizes these data. Statistical analysis revealed a significantly higher rate of LM disease control in patients who received osimertinib than previous generations of EGFR TKI (90.0% (27/30) *vs*. 60.9% (14/23); *p* = 0.012).

**Table 2:** Distribution of our cohort (n = 53) based on treatment regimens received before and following diagnosis of leptomeningeal metastasis (LM).

Pre-LM		Post-LM		
Treatment regimen	n (%)	Treatment regimen	n (%)	LM-disease control (SD/PR); n (%)
First-generation EGFR TKI				
Gefitinib	29 (54.7%)	Gefitinib	1 (1.9%)	0
Erlotinib		Erlotinib	4 (7.5%)	4 (7.5%)
Icotinib		Osimertinib	24 (45.3%)	23 (43.4%)
Second-generation EGFR TKI				
Afatinib	4 (7.5%)	Osimertinib	3 (5.7%)	2 (3.8%)
		None	1 (1.9%)	0
Third-generation EGFR TKI				
Osimertinib	14 (26.4%)	Osimertinib	12 (22.6%)	8 (15.1%)
AZD3759		Non-EGFR TKI	2 (3.8%)	1 (1.9%)
Other TKI				
Pyrotinib	1 (1.9%)	None	1 (1.9%)	0
EGFR TKI-naïve				
Initial treatment	4 (7.5%)	Osimertinib	1 (1.9%)	1 (1.9%)
		Poziotinib	1 (1.9%)	0
		Non-EGFR TKI	2 (3.8%)	1 (1.9%)
Never	1 (1.9%)	Osimertinib	1 (1.9%)	1 (1.9%)

EGFR, epidermal growth factor receptor; LM, leptomeningeal metastasis; SD/PR, stable disease or partial response according to RECIST definitions of response to treatment; TKI, tyrosine kinase inhibitor.

In total, 39 patients (73.6%) received one to 12 cycles of methotrexate as ITC concurrently with other therapies following LM diagnosis. Of these 39 patients, 34 received concurrent EGFR TKI therapy, two patients had chemotherapy, and three patients only received ITC. A total of 31 (79.5%) patients achieved disease control (LM status was improved/stable) with ITC. Among the 31 patients who achieved disease control, 24 (77.4%) received osimertinib concurrent with methotrexate ITC. No significant difference was found in the rate of LM disease control between patients who did and did not receive concurrent ITC after LM diagnosis (79.5% (31/39) *vs*. 71.4% (10/14); *p* = 0.806) and in patients who received osimertinib with or without concurrent ITC (87.5% (21/24) *vs*. 71.4% (10/14); *p* = 0.218).

Nine patients (17.0%) received WBRT following LM diagnosis. Of these nine patients, seven (77.8%) patients achieved disease control with WBRT. No significant difference was found in the rate of LM disease control in patients who did and did not receive WBRT (77.8%, 7/9 *vs*. 77.3%, 34/44; *p* = 0.974).

Taken together, these data suggest that osimertinib therapy is effective in disease control of LM, particularly in patients who developed LM from prior EGFR TKI therapy or in EGFR TKI-naïve patients with LM.

### Survival After Diagnosis With Leptomeningeal Metastasis

**Figure 3:**
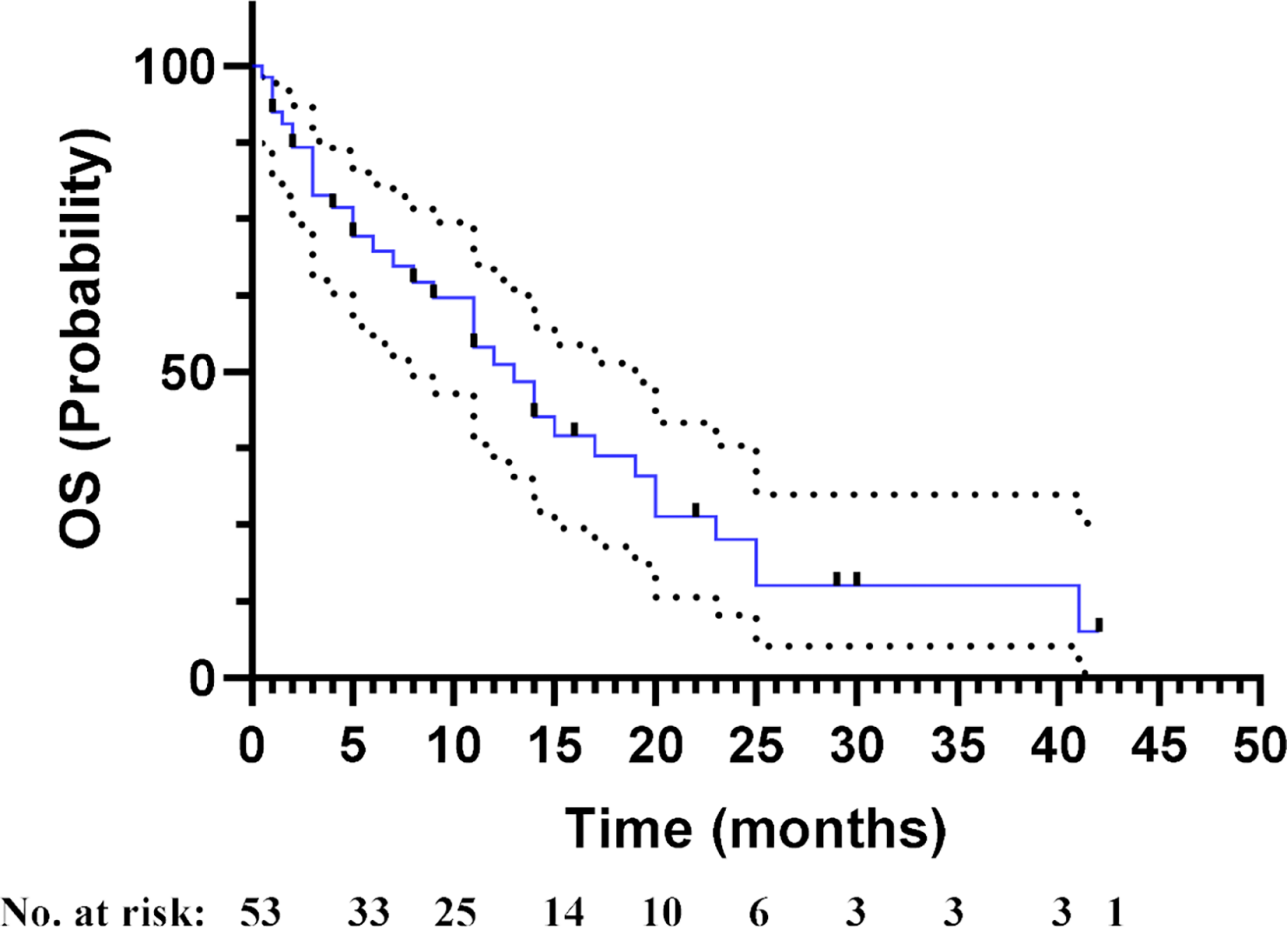
Overall survival (OS) of the 53 patients with *EGFR*-mutated advanced non-small cell lung cancer (NSCLC) with leptomeningeal metastasis (LM) included in this study. Censored data are indicated by tick marks, and 95% CIs are shown by the dotted black lines.

The median OS_LM_ of the patients with *EGFR*-mutated NSCLC included in this study was 13.0 months, ranging from 0.5 to 42.0 months (95% CI = 9.067–16.933), with 64.2% maturity (34/53; **Figure 3**). All 34 patients died due to LM progression.

**Figure 4:**
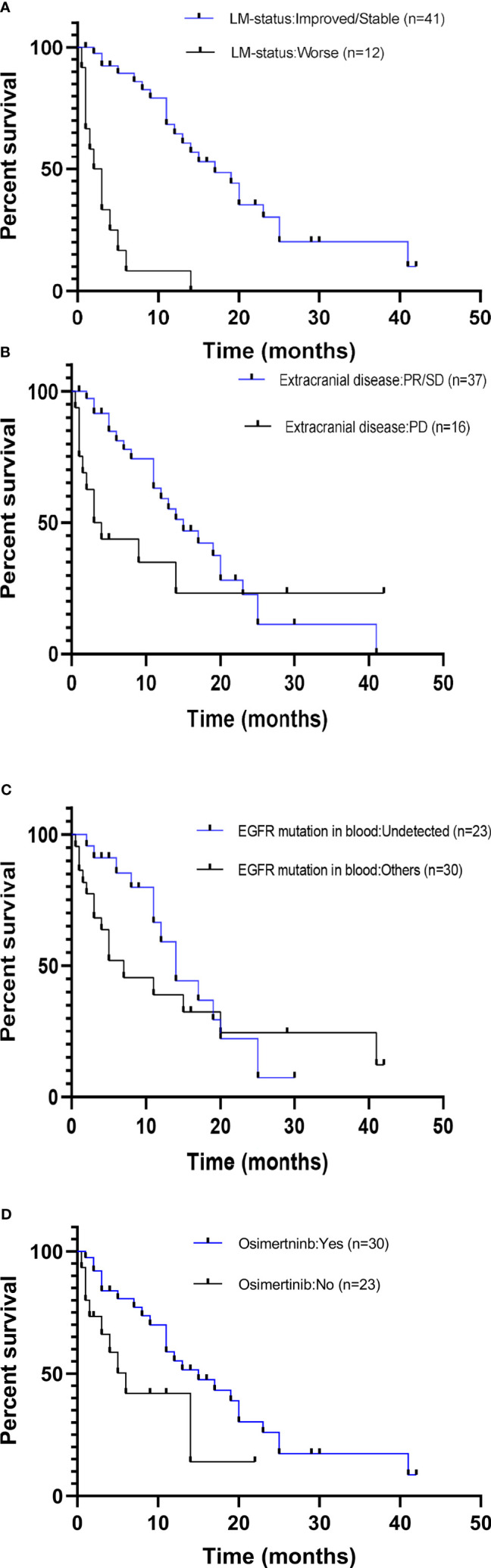
Comparison of overall survival curves for subgroups of patients with *EGFR*-mutated advanced NSCLC with leptomeningeal metastasis (LM). Patients included in this study were subgrouped according to **(A)** LM disease control status (PR/SD *vs*. PD). **(B)** Disease control status of the extracranial metastasis prior to diagnosis with LM (PR/SD *vs*. PD). **(C)** *EGFR* mutation status in the blood at LM diagnosis (positive *vs*. undetected). **(D)** Treatment received following diagnosis with LM (osimertinib *vs*. previous generations EGFR TKI/no treatment). NSCLC, non-small cell lung cancer; PR, partial response; SD, stable disease; PD, progressive disease; EGFR, epidermal growth factor receptor; TKI, tyrosine kinase inhibitor.

**Figure 5:**
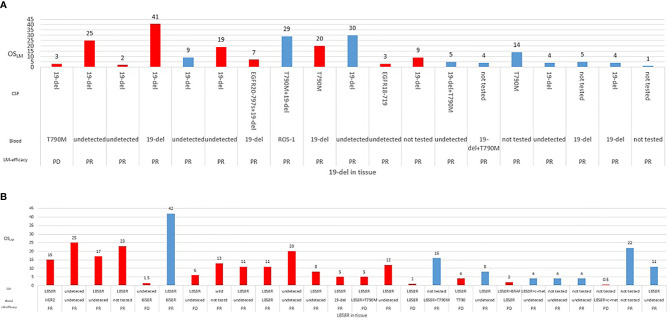
Overall survival of each patient according to *EGFR* mutation status for **(A)** exon 19 deletion (19del) (n = 26) or **(B)** L858R (n = 26) detected from tissue samples. Information for best objective response from treatment received after developing leptomeningeal metastasis (LM), *EGFR* mutations detected from cerebrospinal fluid (CSF), and plasma samples were also indicated.

The potential associations between clinical measures and survival outcomes were compared using univariate analysis, with results shown in **Table 3**. The median OS_LM_ was significantly longer for patients who achieved LM disease control (“improved/stable”) LM status) with treatment regimen received after developing LM than those who had worsening LM status (17.0 *versus* 2.5 months; hazard ratio (HR), 6.800; 95% CI = 3.365–13.740; *p* < 0.001; **Figure 4A**). *EGFR* mutation status in CSF and plasma samples and OS_LM_ data for each patient are shown in **Figure 5**.

**Table 3:** Cox proportional hazard model of factors affecting overall survival.

Factors	Median OS_LM_ (months)	*p*-Value	Hazard ratio	95% CI
Sex (male *vs*. female)	11.0 *vs*. 15.0	0.326	0.73	0.374–1.438
Age (<50 *vs*. ≥50 years)	14.0 *vs*. 13.0	0.916	1.08	0.525–2.209
ECOG PS (1–2 *vs*. 3–4)	11.0 *vs*. 13.0	0.279	0.85	0.413–1.736
Time from diagnosis to LM (≤24 *vs*. >24 month)	13.0 *vs*. 17.0	0.707	0.77	0.379–1.545
LM status (improves/stable *vs*. worse)	17.0 *vs*. 2.5	<0.001	6.80	3.365–13.74
Brain metastasis status (yes *vs*. no)	13.0 *vs*. 12.0	0.590	1.08	0.419–2.799
Extracranial disease status before LM (PR/SD *vs*. PD)	15.0 *vs*. 3.5	0.001	4.29	2.089–8.792
*EGFR* mutation in blood at LM diagnosis (undetected *vs*. others)	14.0 *vs*. 7.0	0.040	2.00	0.965–4.143
*EGFR* mutation in CSF at LM diagnosis (mutation *vs*. undetected)	12.0 *vs*. 13.0	0.775	0.92	0.356–2.397
*EGFR* mutation in tissue before LM (19del *vs*. L858R)	20.0 *vs*. 12.0	0.263	1.67	0.749–3.710
Combined chemotherapy (yes *vs*. no)	14.0 *vs*. 11.0	0.596	1.27	0.527–3.074
Osimertinib therapy after LM diagnosis (yes *vs*. no)	17.0 *vs*. 7.0	0.018	2.43	1.234–4.779
Combined WBRT (yes *vs*. no)	15.0 *vs*. 12.0	0.611	1.25	0.544–2.870
Combined ITC (yes *vs*. no)	13.0 *vs*. 20.0	0.697	0.65	0.283–1.493

19del, exon 19 deletion; CSF, cerebrospinal fluid; ECOG PS, Eastern Cooperative Oncology Group Performance Status; EGFR, epidermal growth factor receptor; ITC, intrathecal chemotherapy; LM, leptomeningeal metastasis; OS_LM_, overall survival after LM diagnosis; PD, progressive disease; PR/SD, partial response/stable disease; WBRT, whole brain radiotherapy.

The median OS_LM_ was significantly longer for patients with extracranial disease categorized as PR/SD at LM diagnosis than those with extracranial disease categorized as PD (15.0 months for PR/SD *versus* 3.5 months for PD; HR, 4.286; 95% CI = 2.089–8.792; *p* = 0.001; **Figure 4B**).

The OS_LM_ was significantly longer for patients whose *EGFR* sensitizing mutations were undetected in blood samples collected at LM diagnosis than those with detectable *EGFR* mutations (17.0 *versus* 7.0 months; HR, 2.000; 95% CI = 0.965–4.143; *p* = 0.040; **Figure 4C**). Among the 14 patients whose extracranial disease was evaluated as PD, only two had undetected *EGFR* mutations in the blood, whereas among the 37 patients whose extracranial disease was evaluated as PR/SD, 21 patients had undetected *EGFR* mutations in their blood at LM diagnosis. There were significantly more patients who had extracranial disease control and have undetected *EGFR* mutations in their blood samples at LM diagnosis (56.8%, 21/37) than those whose extracranial disease was progressive and have undetected *EGFR* mutations in their blood samples at LM diagnosis (14.3%, 2/14) (*p* = 0.007).

The OS_LM_ was significantly longer for patients who received osimertinib than those who received first- or second-generation EGFR TKIs or other non-targeted treatments (15.0 *versus* 6.0 months; HR, 2.429; 95% CI = 1.234–4.779; *p* = 0.045; **Figure 4D**).

Logistic regression analysis showed that LM disease control status was a positive predictive factor for OS_LM_ (odds ratio: 10.797; 95% CI = 4.102–28.419; *p* < 0.001). Sex, age, ECOG PS, BM status, *EGFR* mutation status in CSF, and tissue, combined ITC, WBRT, and chemotherapy received after LM diagnosis, were not significantly associated with OS_LM_ (**Table 3**).

## Discussion

LM is a severe complication of NSCLC and is associated with a low survival rate. Traditional treatments do not improve survival outcomes of patients with *EGFR*-mutated NSCLC who developed LM during EGFR-TKI therapy, and there is still no consensus on the ideal therapeutic strategy that could improve their survival outcomes.

In our study, we observed a median OS_LM_ of 13.0 months in patients with *EGFR*-mutated NSCLC. This finding is similar to that of the subgroup analysis of the AURA study, and longer than the previously reported estimate of 3–10 months (12, 13). We found that the patients who achieved disease control with treatment regimens received after developing LM had significantly longer median OS_LM_ than the patients who were refractory to treatment. LM disease control status was an independent positive predictive factor for overall survival after LM diagnosis. Our findings suggest that, after comprehensive treatment, controlling LM disease is the most effective strategy to prolong the overall survival outcomes of patients with *EGFR*-mutated NSCLC who are diagnosed with LM.

Our findings also demonstrated that the patients with extracranial disease evaluated as PR/SD at LM diagnosis had a median OS_LM_ of 15.0 months, which was significantly longer than the OS_LM_ of patients whose extracranial disease was progressive. The OS_LM_ of patients with undetected *EGFR* mutations in the blood was 14.0 months, which was significantly longer than in patients with detectable *EGFR* mutation (7.0 months). In addition, patients whose extracranial disease was evaluated as PR/SD at LM diagnosis were more likely to have undetected *EGFR* mutations in the blood. The lack of *EGFR* mutations from the blood may indicate a limited concentration of cfDNA released in the circulation that could not be detected by the assay. This lack of *EGFR* mutations and limited concentration of cfDNA in the blood could also be associated with the clinical response of their extracranial disease to the therapeutic regimen received before LM progression. Numerous studies have reported that patients with intracranial malignancies have limited amount of circulating tumor DNA present in the blood, resulting in lower detection of actionable mutations from blood samples of patients with LM (14, 15). CSF, due to direct contact with the central nervous system, are enriched in circulating tumor DNA from LM that could enable profiling of somatic mutations in LM and is the optimal specimen for detecting actionable mutations for patients with LM (14, 15).

The OS_LM_ was significantly longer for patients who received osimertinib after LM diagnosis. Osimertinib, a third-generation EGFR TKI that effectively targets *EGFR*-mutated tumors, including *EGFR* T790M-positive tumors, has been recommended as an effective treatment for patients with *EGFR*-mutated NSCLC. Osimertinib has a beneficial effect on survival, including a longer OS_LM_, owing to its better ability to permeate the blood–brain barrier as compared with previous generations of EGFR TKIs (16). In the BLOOM study, patients with LM who received osimertinib 160 mg once a day had a longer median OS of 11.0 months (95% CI = 8.0–18.0 months) (17). In the AURA study, patients with *EGFR* T790M-positive NSCLC and radiologically diagnosed LM who received osimertinib therapy had a median progression-free survival of 11.1 months and OS_LM_ of 18.8 months (13). In another retrospective study of the AURA cohort, patients with LM who received osimertinib had a significantly longer OS_LM_ of 17.0 months (95% CI = 15.13–18.94), regardless of T790M mutational status as compared with those who did not receive osimertinib (OS_LM_ of 5.5 months; 95% CI = 4.34–6.63) (12). In our study, OS_LM_ was not associated with *EGFR* mutation type (i.e., 19del or L858R mutation) or *EGFR* mutation status in tissue or CSF. Patients with *EGFR*-mutated NSCLC diagnosed with LM may have a longer OS_LM_ when administered with osimertinib after LM diagnosis.

ITC aims to overcome the blood–CSF barrier and has been used in the management of various types of primary solid tumors, including NSCLC (18). The largest study on ITC to date indicated that ITC could significantly prolong the OS_LM_ of patients with NSCLC (17 *versus* 8 weeks, *p* < 0.001) (19). In contrast, some studies have reported no significant OS_LM_ benefit for patients with NSCLC who received ITC compared with those who did not (3, 20, 21). In our study, we did not observe any improvement in the rate of LM disease control and OS_LM_ in patients who received ITC. At present, methotrexate is the only available drug for ITC, and the selection of available drugs is limited. Phase I/II clinical studies have reported promising safety and efficacy outcomes for intrathecal pemetrexed for patients with *EGFR*-mutant LM-NSCLC (22, 23). A clinical study reported clinical response rate of 84.6% (22/26) and median overall survival of 9.0 months (n = 30; 95% CI = 6.6–11.4 months) for patients who received intrathecal pemetrexed (22). Phase III clinical trials are expected to provide evidence for more ITC drug selection and clinical benefits for patients with LM in the future.

Traditional systemic chemotherapy is another therapeutic option for NSCLC patients diagnosed with LM. Owing to differences in treatment history prior to LM diagnosis, blood–brain barrier penetrability, pathological NSCLC subtype, and molecular profile, there is still a lack of standardized, effective chemotherapy treatment regimens for patients with LM. The use of pemetrexed after LM diagnosis has been reported to provide significantly longer post-LM survival for patients with *EGFR*-mutant NSCLC and LM (21). As compared with erlotinib alone, a combination of erlotinib and pemetrexed/cisplatin was reported to improve intracranial PFS and has been suggested as an effective therapeutic option in treatment-naïve patients with lung adenocarcinoma with BM (9 *versus* 2 months, *p* = 0.027) (24). At present, there is a lack of evidence from randomized controlled trials on the clinical outcomes of targeted therapy combined with cytotoxic chemotherapy in NSCLC patients after first-line treatment with EGFR TKIs.

WBRT is mainly used for the management of patients with concurrent BMs. Several studies have suggested that WBRT could improve clinical outcomes in patients with NSCLC with LM (25, 26), but other studies have shown no survival benefit associated with WBRT in this group (4, 21). In our study, we also did not observe any survival benefit with WBRT. There is currently a lack of evidence for radiotherapy as an effective treatment for patients with NSCLC and LM from randomized controlled trials. In addition, whole spinal cord radiotherapy is highly toxic and is associated with high mortality (27). Further studies are needed to investigate the role of radiotherapy in this group.

Our study has several limitations. Our study only included a small cohort in a single institution that could introduce sample bias. The diversity and complexity of treatment methods may have affected the clinical outcomes analyzed in this study. Thus, future studies must continue to overcome these methodological challenges when assessing clinical efficacy.

In conclusion, our study provided real-world clinical evidence that patients with *EGFR*-mutated NSCLC, particularly those who progressed from previous generations of EGFR TKI, had better clinical outcome and significantly longer survival outcome with osimertinib treatment. Our findings also suggest that intracranial and extracranial disease control is the most effective strategy to prolong the overall survival outcomes of these patients.

## Data Availability Statement

The original contributions presented in the study are publicly available. This data can be found here: https://ngdc.cncb.ac.cn/gsa-human/; HRA001818 (https://bigd.big.ac.cn/gsa-human/browse/HRA001818).

## Ethics Statement

The studies involving human participants were reviewed and approved by the ethical committee of the Cancer Hospital, Chinese Academy of Medical Sciences, and Peking Union Medical College. The patients/participants provided their written informed consent to participate in this study.

## Author Contributions

All the authors were involved in the conception and design of the study, data collection, data analysis, manuscript writing, editing, and approving the manuscript.

## Funding

This work was supported by grants from the Sisco pilot Cancer Research Fund (supplementary) project (grant number Y-2019AZQN-1060). The funders had no role in the conceptualization, design, data collection, analysis, decision to publish, or preparation of the manuscript.

## Conflict of Interest

The authors declare that the research was conducted in the absence of any commercial or financial relationships that could be construed as a potential conflict of interest.

## Publisher’s Note

All claims expressed in this article are solely those of the authors and do not necessarily represent those of their affiliated organizations, or those of the publisher, the editors and the reviewers. Any product that may be evaluated in this article, or claim that may be made by its manufacturer, is not guaranteed or endorsed by the publisher.
